# Interrogating Synaptic Architecture: Approaches for Labeling Organelles and Cytoskeleton Components

**DOI:** 10.3389/fnsyn.2019.00023

**Published:** 2019-08-23

**Authors:** Sofiia Reshetniak, Silvio O. Rizzoli

**Affiliations:** ^1^Institute for Neuro- and Sensory Physiology, Center for Biostructural Imaging of Neurodegeneration (BIN), University Medical Center Göttingen, Göttingen, Germany; ^2^International Max Planck Research School for Molecular Biology, Göttingen, Germany

**Keywords:** synapse, vesicles, cytoskeleton, actin, nanoscopy, super-resolution

## Abstract

Synaptic transmission has been studied for decades, as a fundamental step in brain function. The structure of the synapse, and its changes during activity, turned out to be key aspects not only in the transfer of information between neurons, but also in cognitive processes such as learning and memory. The overall synaptic morphology has traditionally been studied by electron microscopy, which enables the visualization of synaptic structure in great detail. The changes in the organization of easily identified structures, such as the presynaptic active zone, or the postsynaptic density, are optimally studied via electron microscopy. However, few reliable methods are available for labeling individual organelles or protein complexes in electron microscopy. For such targets one typically relies either on combination of electron and fluorescence microscopy, or on super-resolution fluorescence microscopy. This review focuses on approaches and techniques used to specifically reveal synaptic organelles and protein complexes, such as cytoskeletal assemblies. We place the strongest emphasis on methods detecting the targets of interest by affinity binding, and we discuss the advantages and limitations of each method.

## Introduction

**FIGURE 1:**
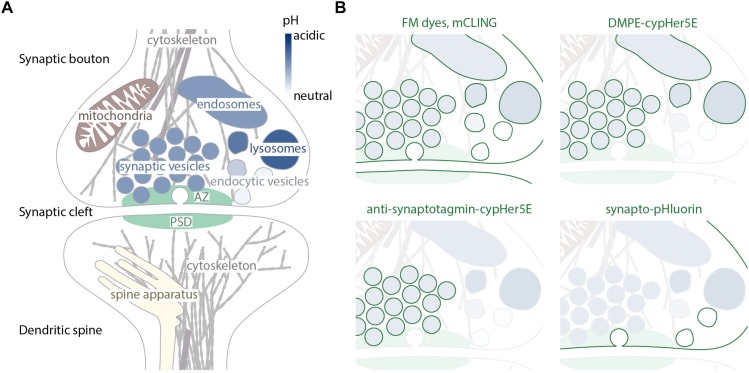
Synaptic organelles and specificity of probes directed toward recycling membranes. **(A)** Schematic representation of main organelles present at the synapses. The pH level of endosomal components is visualized with different shades of blue. The different pHs aid in differential labeling by membrane probes, as shown in **(B)**. **(B)** Membranes that are labeled by different membrane-labeling tools highlighted in green, the identity of the organelles preserved from **(A)**.

Chemical synapses support neurotransmission by releasing neurotransmitter from the presynaptic side, and responding to it on the postsynaptic side. The presynaptic bouton, or terminal, has here a highly dynamic role, since it responds to activation via plasma membrane depolarization by forcing the fusion of synaptic vesicles to the membrane, which is followed by the diffusion of the neurotransmitter molecules to postsynaptic receptors. The synaptic vesicles, defined as small organelles with an outer diameter of approximately 40 nm which contain neurotransmitters and fuse to the plasma membrane upon stimulation (Schikorski and Stevens, 1997), are highly enriched in synaptic boutons, and are, in principle, not functional elsewhere. Along with vesicles, the boutons also contain several other organelles that are not necessarily specific for synapses, such as endosome-like structures (with which synaptic vesicles as well as other endocytic vesicles fuse and where, presumably, their cargo undergoes sorting) (Heuser and Reese, 1973), ribosomes (Crispino et al., 1997), smooth endoplasmic reticulum (ER), and mitochondria (Lysakowski et al., 1999; Figure 1A). While the constant presence of endosomes and components of the protein-synthesizing and -sorting machineries (endoplasmic reticulum, ribosomes) in pre-synaptic terminals throughout synaptic development is still heavily debated (Akins et al., 2009), the presence of mitochondria here has been well-established since the first electron microscopy observations of

synapses (Palay, 1956). Large mushroom spines tend to contain a specialized compartment, composed of multiple membrane stacks, known as the spine apparatus (Spacek and Harris, 1997; Figure 1A).

Apart from synaptic vesicles, two non-membrane bound structures can be considered synapse-specific organelles: the active zone and the post-synaptic density (Figure 1A). The active zone (AZ) of presynaptic terminals contains multiple proteins, including molecules involved in cellular adhesion, voltage-gated calcium channels, scaffold proteins, and multiple exocytosis co-factors. The AZ proteins regulate the docking, priming and fusion of synaptic vesicles (Südhof, 2012). AZs are apposed to post-synaptic densities (PSDs), which are protein-rich structures containing adhesion molecules, neurotransmitter receptors, adaptors (such as the PSD95 family proteins), and signaling proteins (Kaizuka and Takumi, 2018).

Cytoskeletal proteins found in the synapses include tubulin, actin, and septin (Wilhelm et al., 2014). Microtubules are known to form bundles along axons and bind presynaptic mitochondria (Chan and Bunt, 1978; Perkins et al., 2010; Graffe et al., 2015), synaptic vesicles (Bird, 1976), and to be positioned close to the plasma membrane and the active zone in the synaptic boutons (Gordon-Weeks et al., 1982). Actin is the most predominant component of the cytoskeleton and in presynaptic terminals two distinct populations of actin filaments are described. First, F-actin was shown to be a component of the active zone cytomatrix (Bloom et al., 2003), where it may form a barrier for vesicle release (Morales et al., 2000). Second, it has been also shown to surround synaptic vesicle clusters (Sankaranarayanan et al., 2003; Richards et al., 2004), where it is thought to contribute to vesicle recycling. In post-synapses actin forms a network of long linear and short branched filaments (Korobova and Svitkina, 2009) that reach the PSD where they may stabilize postsynaptic proteins (Allison et al., 1998; Kuriu et al., 2006).

Many synaptic components were discovered and studied using electron microscopy. The main advantage of this technique is its high resolving power that allows examining fine structures with nanometer precision. A crucial drawback, however, is its inability to reveal the identity of the structures. This has been addressed by labeling structures of interest using gold-conjugated antibodies raised against target proteins (immunoelectron microscopy) but such stainings often result in relatively low labeling densities.

A widely used approach to specifically visualize cellular components is to use genetically encoded fluorescent tags [such as green fluorescent protein (GFP) and its derivatives] fused to proteins of interest with consequent imaging with fluorescent microscopy. This requires protein overexpression or genome editing but results in a high labeling density, and enables live cell imaging of tagged molecules. The common problem associated with such an approach is impaired targeting or trafficking of tagged proteins, which can lead to a different behavior, and different subcellular localization, for the chimeric proteins when compared to native ones.

Nevertheless, the properties of most fluorescent proteins, in terms of intensity or stability during imaging, are sub-optimal, when compared to chemical dyes. This has raised substantially the interest in fluorescent probes that specifically bind to molecules of interest, and that can be conjugated to specific chemical dyes. Such elements are commonly used to visualize endogenous cellular components at their native locations, and enable investigators to exploit recent advances in super-resolution microscopy, thereby combining the two main advantages of the methods described above: labeling of cellular components with high specificity and efficiency, and nanometer resolution (Hell and Wichmann, 1994; Hofmann et al., 2005; Rust et al., 2006; Sharonov and Hochstrasser, 2006). Fluorescently labeled antibodies are the most commonly used tool in this approach, but many other probes have been developed for labeling of different cellular organelles and components. In the following section we will discuss the most prominent ones, their mechanisms of action, main advantages, and disadvantages.

## Materials and Methods

### Super-Resolution Microscopy Techniques

The probes we will discuss here were developed to be used in combination with light microscopy to specifically visualize certain organelles and structures. However, conventional imaging techniques have a significant disadvantage of not being able to resolve objects that are positioned closer than ∼200 nm to each other, due to the diffraction limit. Two types of approaches have been developed to overcome the diffraction barrier. First, the coordinate-targeted approach, which uses a patterned light beam to determine the coordinates from which fluorophores are permitted to emit. This approach is used by the stimulated emission depletion microscopy family (STED; Hell and Wichmann, 1994), and the saturated structured illumination microscopy family (SIM; Gustafsson, 2005). SIM currently reaches resolutions of ∼60–100 nm, while most STED applications in biology reach ∼40–50 nm. Second, the single-molecule based approach, which is based on the determination of the positions of single fluorophores that are allowed to emit randomly. This approach is typical of concepts such as photo-activated localization microscopy (PALM; Betzig et al., 2006), stochastic optical reconstruction microscopy (STORM and dSTORM; Rust et al., 2006; van de Linde et al., 2011), or ground state depletion microscopy followed by individual molecule return (GSDIM; Testa et al., 2010). This approach can reach a higher spatial resolution, typically of ∼20–30 nm in biological samples. Even higher resolution has been obtained with the MINFLUX concept (maximally informative luminescence excitation; Balzarotti et al., 2017). This technique combines a coordinate-targeted approach, such as used in STED microscopy, with single-molecule localization, as in PALM or STORM, and enables resolutions of ∼1–4 nm. Finally, super-resolution can also be achieved through physically expanding the specimen after embedding into a swellable gel (Chen et al., 2015). Resolutions of ∼20–70 nm have been attained with this approach (Chen et al., 2015; Chang et al., 2017; Truckenbrodt et al., 2018a).

### Visualizing Synaptic Organelles Using Non-specific Membrane-Labeling Tools

**FIGURE 2:**
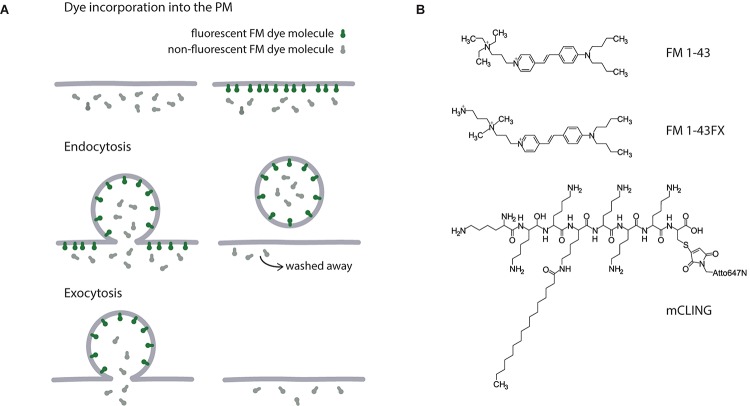
Tools for visualizing recycling vesicles. **(A)** The mechanism of styryl dyes action. Colored and gray shapes represent fluorescent and non-fluorescent FM molecules, respectively. Upon addition to cellular medium, FM dye incorporates into the outer leaflet of the PM and becomes fluorescent. Following endocytosis, these fluorescent molecules are trapped in recycling vesicles, while unspecific signal from the PM can be eliminated by washing the cells. Since FM dye incorporation into the membrane is reversible, after the vesicle is fused with the PM during exocytosis, the fluorescent signal is lost again. **(B)** Chemical structures of FM 1–43, FM 1–43FX, and mCLING.

With synaptic vesicles and endosomes being the most prominent and important organelles of the pre-synapse, many tools exists for their visualization (Figure 1B). These labels are often hydrophobic molecules capable to incorporate into or permeate the plasma membrane. One classical example is styryl dyes such as FM 1–43. They are molecules that are highly fluorescent in a hydrophobic environment of cellular membranes and have significantly lower quantum yield in aqueous solutions such as extracellular medium (Betz et al., 1992). Their ability to reversibly incorporate into outer leaflet of the plasma membrane but not to penetrate it makes them a perfect tool to study endo- and exocytosis in live cells. Upon addition to cells, FM dye molecules incorporate into the plasma membrane and some of them get internalized via endocytosis. When cells are washed and all FM dye molecules that remained in the outer leaflet of the plasma membrane are gone, the only source of fluorescence is the internalized vesicles whose recycling can be now followed by fluorescence microscopy. When these vesicles undergo exocytosis following stimulation, the fluorescence is lost again due to FM dye leaving the membrane for the aqueous extracellular solution (Figure 2A).

An interesting application of FM dyes has exploited the different molecular structures of these probes. Both FM 1-43 and FM 2-10 are green dyes, but the former is a larger molecule, and inserts more strongly into membranes. This renders it more difficult to wash from synaptic membranes (Pyle et al., 2000; Richards et al., 2000). A comparison between the wash-off (destaining) kinetics of the dyes can reveal endocytosis intermediates, such as infoldings, that remain open on the plasma membrane after stimulation. Such intermediates lose FM 2–10, as this dyes is easily washed off, but not FM 1–43 (Richards et al., 2000). The FM 1–43 retained in vesicles or in endocytosis intermediates can be quenched by adding to the buffers small molecules such as bromphenol blue (Harata et al., 2006), thereby providing further information on the different vesicle recycling modalities.

In addition to fluorescent properties, FM dyes can be used in electron microscopy due to their ability to undergo photoconvertion (or photo-oxydation). Upon strong illumination in the presence of diaminobenzidine, a dark precipitate is formed where FM dye is located making it visible in electron microscopy. This allows even higher resolution for imaging of structures labeled with FM dye (Denker et al., 2009, 2011; Hoopmann et al., 2012).

The chemical structure of FM dyes does not allow them to be fixed by aldehyde-based fixatives, which renders it problematic to use them in combination with immunostainings (Figure 2B). They are often lost from trafficking organelles, and can even be trapped in other cellular compartments after fixation (Revelo et al., 2014). Fixable variants of FM dyes containing single amine functional groups were developed to overcome this difficulty (e.g., FM 1–43FX), but they are still poorly fixed by common fixatives, and are also not optimized for use in super-resolution microscopy. These problems have beed solved with development of the membrane-binding fluorophorecysteine-lysine-palmitoyl group (mCLING). mCLING consists of a fluorophore bridged to a palmitoyl tail by an octapeptide. Six lysines of the peptide allow the probe to be fixed by aldehydes thus preventing its loss from the membrane or mislocalization post-fixation (Revelo et al., 2014).

Both styryl dyes and mCLING have a common disadvantage: they are not specific for any particular organelle and stain all vesicles that undergo recycling of membranes, as well as the plasma membrane (Figure 1B). This issue can be partially solved by usage of lipid-based pH-sensitive labels. Dyes such as cypHer5E are highly fluorescent in acidic environments and are quenched at a neutral pH. If conjugated to phospholipids they can get incorporated into the plasma membrane just like FM dyes, but remain non-fluorescent there. Only after the dye is internalized and reaches a lumen of an acidic organelle such as a late endosome or a synaptic vesicle, it becomes fluorescent allowing visualization of the organelle (Kahms and Klingauf, 2018). As soon as the vesicle undergoes exocytosis exposing the pH-sensitive dye to a neutral environment of the extracellular fluid, the fluorescence disappears again. While allowing the investigator to differentiate between the plasma membrane, coated vesicles, and endo-lysosomal system or synaptic vesicles, this approach is unable to distinguish different organelles that have the same luminal pH (Figure 1B).

One note of caution in using lipid dyes that insert in the plasma membrane, as the ones presented here, is that they could, in principle, affect membrane tension, and may therefore influence synaptic vesicle fusion. One study suggested this for FM 4–64 (Zhu and Stevens, 2008), by comparing synaptic release in presence and in absence of the dye. However, the effects noted were mild, and could also be attributed to a Förster Resonance Energy Transfer (FRET) effect taking place between the green reporter used here to measure exocytosis and the red FM 4–64. Overall, this suggests that such dyes are relatively “safe” tools to use for synaptic investigations, albeit one should aim to use low concentrations. Low concentrations are also useful in reducing phototoxicity. The FM dyes have a low photostability, which renders them excellent tools for photo-oxydation, as mentioned above, but reduces their applicability to long-term live imaging. Complex live imaging experiments, such as measurements of single-vesicle dynamics, can be performed (Zenisek et al., 2000, 2002), but long imaging periods should be avoided, especially as photodamage to the cells takes place several minutes before significant photobleaching can be observed, in our experience.

### Increasing Specificity by Using Antibodies Directed to Epitopes Exposed on the Cell Surface

**FIGURE 3:**
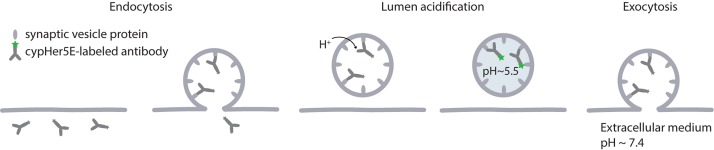
CypHer5E as a tool to visualize the synaptic vesicle cycle. CypHer5E is a pH-sensitive fluorophore that can be coupled to antibodies against the luminal domain of synaptic vesicle proteins. Following exocytosis, the luminal domains of the antibodies are exposed to the extracellular medium and the antibodies can bind them. At the neutral pH of the extracellular medium, the fluorophore is quenched. When the luminal pH is lowered after endocytosis, the fluorophores bound to the synaptic vesicle proteins through respective antibodies become fluorescent and allow visualization of internalized vesicles. When such a vesicle fuses with the plasma membrane during synaptic activity, the fluorescence is lost again.

To specifically visualize particular organelles one can turn to affinity tools. A classical approach of labeling synaptic vesicles is use of fluorescently labeled antibodies against luminal domain of synaptotagmin (Matteoli et al., 1992). High specificity and affinity of antibodies to the target proteins ensure specific labeling of desired organelles even after they undergo membrane recycling. Usage of antibodies against a luminal domain of the protein allows tracking vesicle trafficking in live cells, as the antibodies can be added to the cellular medium and internalized via endocytosis. The antibodies can be coupled to various fluorophores to fit requirements for specific experiment and microscopy method used. For example, the pH-sensitive dye cypHer5E, which we described above, can be used to follow synaptic vesicle when coupled to a luminal domain of a synaptic vesicle protein synaptotagmin (Figures 1B, 3). Since cypHer5E is only fluorescent in the acidic environment of synaptic vesicles and is quenched at the neutral pH of extracellular medium, it can specifically reveal the exocytosis of synaptic vesicles when bound to a synaptic vesicle protein (Martens et al., 2008; Hua et al., 2011). It is important to note, however, that some antibodies might affect protein distribution and trafficking in live cells. Thus, rabbit polyclonal antibodies against the luminal domain of synaptotagmin 1 have been suggested to alter synaptic function (Afuwape et al., 2017). At the same time, mouse antibodies against the same target, which are usually used for vesicle tracking experiments (Matteoli et al., 1992; Kraszewski et al., 1995; Sara et al., 2005; Fernández-Alfonso et al., 2006; Wienisch and Klingauf, 2006; Hua et al., 2010), have not been reported to have such an effect, and do not perturb vesicle trafficking even when used for several days (Truckenbrodt et al., 2018b).

**FIGURE 4:**
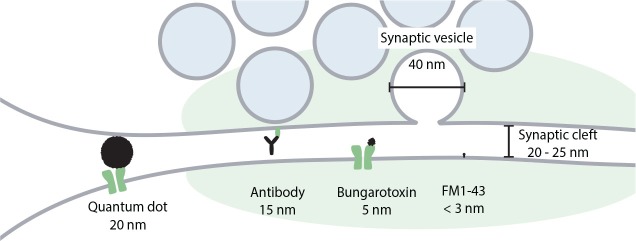
Comparison of the size of quantum dots with synaptic structures and other probes used to label the plasma membrane, recycling membranes, or membrane receptors in the synapse. Realistic sizes are presented for all labels. For quantum dots we assume that their core is covered by a streptavidin layer, to which antibodies are then attached.

Strong and selective binding of antibodies to the target proteins makes them also a useful tool for long-term imaging. When labeled with a bright and photo-stable reporter such as a quantum dot, they can be used for prolonged observation of organelles (Park et al., 2012) or even single molecules such as postsynaptic receptors (Dahan et al., 2003; Groc et al., 2004). To visualize the organelles, quantum dots must be coupled to the antibodies against the luminal domains of the synaptic vesicle proteins, as in the case of cypHer5E-labeled antibodies. This is often achieved through usage of biotinylated secondary antibodies and streptavidin-coated quantum dots. Alternatively, quantum dots can be directly coated with secondary antibodies. When the luminal domain of the synaptic vesicle protein faces the extracellular medium after exocytosis, the antibodies and the quantum dots can label the inside of the vesicle. They are then internalized together with the target protein, resulting in the newly formed synaptic vesicle being loaded with the quantum dot. This has been used to visualize endocytosis (Hoopmann et al., 2010) as well as single exocytic events (Zhang et al., 2009; Park et al., 2012). To track plasma membrane proteins, quantum dots are coupled to antibodies against extracellular domains of the target proteins. This allows following diffusion of single post-synaptic receptors in the plasma membrane of a live neuron (Tardin et al., 2003; Howarth et al., 2005; Bannai et al., 2006; Chang et al., 2012; Wang et al., 2016; Taylor et al., 2018). Physical and optical properties of quantum dots allow the observation of the labeled structures for minutes without considerable photobleaching, what is usually difficult to achieve with the use of most organic dyes and especially genetically encoded fluorophores such as GFP. Additionally, since quantum dots are electron-dense, they can, in principle, be used for the visualization of the structure of interest in the electron microscopy. By their nature, quantum dots are hydrophobic and also toxic to the cell, so have to be coated with shells of polar substances to make them water-soluble and compatible with biological specimens (Dubertret et al., 2002). In addition to these shells, quantum dots have to be covered with streptavidin and antibodies layers, increasing their size substantially. While the fluorescent core might be as small as 2 nm, the total size of the label can reach 20–40 nm (Michalet et al., 2005), which is comparable with the width of the synaptic cleft and the synaptic vesicle diameter (Figure 4). This limits quantum dots’ ability to penetrate synaptic cleft, resulting in labeling of mainly extrasynaptic population of membrane proteins, and can affect diffusion of the labeled proteins (Lee et al., 2017; Delgado and Selvin, 2018). Hence, special care must be taken when designing an experiment with the use of quantum dot labeling, to ensure that the quantum dots are of a suitable size to effectively label proteins in the desired compartment, and to avoid causing significant changes to the location and trafficking of the proteins.

### Identifying Organelles by Specific Cell-Permeable Labels

All probes discussed above bind at the outer surface of the plasma membrane and must be endocytosed to label organelles of interest, where they remain attached to the luminal surface of the membrane. This approach cannot be used to visualize organelles that are not involved in direct membrane exchange with the plasma membrane, such as lysosomes or mitochondria. Cell-permeable labels that accumulate in the organelles of interest were developed to label these.

To label lysosomes, acidotropic dyes such as neutral red, acridine orange, DAMP (N-(3-[2,4-dinitrophenyl amino] propyl)-N-(3-aminopropyl)methylamine), and LysoTracker can be used. They are able to penetrate cellular membranes, but after getting protonated at acidic pH of lysosomes they lose this ability and are unable to escape the organelle (Wiederschain, 2011). Similarly to pH-sensitive dyes, most acidotropic molecules cannot discriminate between different organelles and accumulate in any organelle that has low pH. Additionally, DAMP is not fluorescent, and therefore has to be visualized by fluorophore-coupled antibodies, thus making it unsuitable for live cell studies. LysoTracker^®^ (Thermo Fisher Scientific) is the most commonly used tool for labeling lysosomes and is commercially available in various colors, making it suitable for multi-color imaging. While LysoTracker can be used for live imaging, it induces lysosomes’ alkylation following longer incubation periods and thus can only be used for shorter periods of time. Similar to LysoTracker, LysoSensor^TM^ probes (Thermo Fisher Scientific) also accumulate in acidic organelles, but, additionally, they also exhibit changes in fluorescence intensity in reaction to changes in pH, making it possible to observe lysosomes dynamic and biogenesis (Diwu et al., 1999).

For labeling of mitochondria, membrane-potential-dependent dyes such as rhodamine 123, tetramethylrhodamine methyl ester (TMRM), and tetramethylrhodamine ethyl ester (TMRE) can be used. They are cell-permeable dyes that accumulate in mitochondria in response to mitochondrial transmembrane potential. These dyes are highly fluorescent in the inner mitochondrial membrane but are self-quenched in mitochondrial lumen. Since their retention in mitochondria depends on the membrane potential, they can only be sequestered by active mitochondria and are washed away from dead and fixed cells (Scaduto and Grotyohann, 1999). MitoTracker^®^ (Thermo Fisher Scientific) is a similar label that has an additional chloromethyl moiety, which reacts with thiols in mitochondria keeping MitoTracker associated with mitochondria even after fixation (Poot et al., 1996).

Similar membrane-permeable probes for labeling of endoplasmic reticulum have also been developed. Commercially available ER-Tracker^TM^ Green and Red (Thermo Fisher Scientific) contain glibenclamide moieties that bind to the sulfonylurea receptors of ATP-sensitive K^+^ channels (Hambrock et al., 2002), commonly found in ER (Smith et al., 2007), while ER-Tracker Blue-White DPX selectively labels ER through an unexplained mechanism. All of these labels penetrate cellular membranes and have been used to image ER in live neurons (Bannai et al., 2004; Choi et al., 2006; Gallego-Sandín et al., 2009; Tucker et al., 2016). Another probe, called ER Thermo Yellow enables ER staining in live and fixed cells and, in addition, enables monitoring temperature changes inside ER (Arai et al., 2014). Fluorescent flavonoids have been also shown to be a potential tool for ER visualization with minimal toxicity (McDonald et al., 2016), but have gained little popularity so far. Finally, NH_2_-BODIPY is a new probe that can be used for labeling of ER in both live and fixed cells and imaged with STED microscopes (Sekhar et al., 2019), providing a valuable option for super-resolution studies of ER in fixed cells, without the need to express ER markers fused to fluorescent proteins or immunostainings.

### Visualisation of Other Synapse-Specific Structures

All discussed above probes for the visualization of membranous compartments rely on binding to epitopes as they are exposed during the fusion of the compartments to the plasma membrane, or become trapped in the respective compartments due to their specific transmembrane potentials or luminal pH values. Synapse-specific structures from the cytosol, such as the AZ and PSD, cannot be labeled by a similar approach, and their visualization remains limited to the use of antibodies or GFP chimeras. AZs are often visualized by labeling scaffold proteins RIM1, Piccolo and Bassoon (Dani et al., 2010; Nishimune et al., 2016; Schoen et al., 2016; Truckenbrodt et al., 2018a; Heller et al., 2019). In the case of PSD, the most commonly targeted soluble proteins are scaffolds PSD-95, Shank and Homer proteins. By employing super-resolution imaging and antibody stainings or fluorescent protein fusions, they can be visualized in fixed or live cells to report the localization and organization of the PSD (Dani et al., 2010; MacGillavry et al., 2013; Tao-Cheng et al., 2014; Broadhead et al., 2016). Other commonly labeled epitopes include the cytosol-exposed or extracellular domains of neuroligins and of postsynaptic AMPA, NMDA, GABA, and Glycin receptors. They can be labeled by antibodies, or monomeric streptavidin [when tagged with a biotinylation substrate peptide (Liu et al., 2013; Chamma et al., 2016a; Lee et al., 2017)]. Many can be also targeted in live cells (Schapitz et al., 2010; Ladepeche et al., 2013; Liu et al., 2013; Nair et al., 2013; Specht et al., 2013; Bannai et al., 2015; Chamma et al., 2016a; Jézéquel et al., 2017; Lee et al., 2017; Mikasova et al., 2017; Patrizio et al., 2017; Choquet, 2018; Haas et al., 2018). ER in dendritic spines can be revealed if actin binding protein synaptopodin, which is also known to be associated with the spine apparatus, is targeted by fluorescent protein fusion or immunostaining (Mundel et al., 1997; Deller et al., 2000; Orth et al., 2005; Vlachos et al., 2009). In addition to antibodies, an increasing selection of smaller probes, including nanobodies (Vincke and Muyldermans, 2012), becomes available for improved imaging of synaptic proteins with super-resolution microscopy. Nanobodies against SNAP25, syntaxin 1, Homer 1, gephyrin, alpha-synuclein, vGLUT and several other proteins have been developed (Lynch et al., 2008; Schenck et al., 2017; Dong et al., 2019; Maidorn et al., 2019), enabling labeling of these proteins in cells without the need to overexpress them.

### Probing Synaptic Structure by Using Natural or Chemically Derived Toxins

An alternative to antibodies that allows very specific recognition and hence makes effective labeling of target proteins in the synapse possible is neurotoxins. Naturally used by venomous predators to paralyze or kill their prey as quick as possible, neurotoxins evolved to bind strongly and highly selectively to their targets, making them a useful tool for visualization of these proteins. Some neurotoxins and their chemical analogs have been used for investigation of postsynaptic receptors for decades (Adams and Olivera, 1994). One such an example is bungarotoxin – a short protein toxin found in the venom of snakes from the genus *Bungarus*. Kappa-bungarotoxin is a variant specific to nicotinic acetylcholine receptors in neurons, which, when appropriately labeled, can reveal localization of the acetylcholine receptors in the post synaptic terminals (Chiappinelli, 1983). Fluorescently labeled alpha-bungarotoxin is commonly used for imaging of the alpha-subunit of the nicotinic acetylcholine receptor in neuromuscular junctions (Anderson and Cohen, 1977; Borodinsky and Spitzer, 2007; Fricker et al., 2013) and is commercially available in variety of colors from different manufacturers. It has also been shown to bind the acetylcholine receptor in post-synapses of neurons (Colquhoun and Patrick, 1997) and used for imaging of the receptors *in vivo* (McCann and Lichtman, 2008). Besides acetylcholine receptors, alpha-bungarotoxin was also used for studying AMPA receptor and GABA receptors localization and trafficking in neurons (Sekine-Aizawa and Huganir, 2004; Wilkins et al., 2008; Brady et al., 2014). In the latter studies, the alpha-bungarotoxin-binding site was fused to the proteins of interest, to enable the toxin to recognize receptors it usually does not bind to. This makes the bungarotoxin-binding site an affinity tag which, in principle, can be used for specific visualization of any membrane protein as long as adding this tag does not change the receptor targeting and trafficking.

Another group of neurotoxins that started to be used for postsynaptic receptor visualization more recently is conotoxins – small peptides of 10–30 amino acids found in the venom of the *Conum* snails. Various types of conotoxins were identified, each having a high affinity to a different target protein, including nicotinic acetylcholine receptors (Nicke et al., 2004), voltage-gated sodium channels (Leipold et al., 2005), potassium channels (Shon et al., 1998), and calcium channels (Nielsen et al., 2000). These small peptides can be conjugated chemically to fluorescent dyes and used as small probes to label respective proteins (Vishwanath and McIntosh, 2006). Very similar in structure, a component of deathstalker scorpion venom chlorotoxin has high affinity for chloride channels (DeBin et al., 1993). Many other scorpion venom components are used to study channels and receptors and can also be produced as recombinant fluorescent proteins to be used in microscopy (Kuzmenkov et al., 2016). While these toxins provide very high affinity and specificity, working in nano- and picomolar concentrations and being able to distinguish between very similar classes of receptors, their small size often makes it difficult and expensive to label them with fluorescent reporters, thus limiting their use.

### Labeling Proteins With Small Affinity Tags

When no specific binder exists for a target protein, and fusion with a fluorescent protein has to be avoided, small peptide tags can be used to specifically visualize such proteins. They are short sequences of several amino acids that can be fused to any protein of interest and then targeted by a strong specific binder. The FLAG-, HA-, and myc-tags (Evan et al., 1985; Hopp et al., 1988; Wilson et al., 2005) are ones of the most commonly used affinity tags in imaging. Due to their small size (∼1.1 kDa) they are not expected to drastically affect the proteins’ traffic or function, and can be visualized by any imaging method following a staining with antibodies labeled with a suitable fluorophore. To increase the brightness of labeling, several copies of one tag can be fused to a protein, resulting in several antibodies binding to one target. When expressed on the extracellular domains of the plasma membrane proteins, these tags can be used for live cell imaging and tracking, as in the case of discussed above bungarotoxin-binding sites. However, the bivalency of the antibodies might introduce artifacts caused by protein clusters formation. The large size of the antibodies also restricts their ability to penetrate into confined and crowded environments, and can affect protein trafficking when applied to live cells. To solve these issues, smaller monovalent binders can be used. One possible alternative is monomeric streptavidin (Chamma et al., 2016a). To be recognized by streptavidin, the target protein must be fused to a 15 amino acid biotinylation substrate peptide (AP-tag). When biotin and biotin ligase are added to the cellular medium, the AP-tag is biotinylated and can be specifically bound by streptavidin. In addition to having an advantage of not cross-linking the target proteins, monomeric streptavidin is also substantially smaller in size compared to antibodies or monovalent streptavidin, decreasing the influence of large label on protein trafficking. This approach has been used to visualize several synaptic proteins including neuroligin, neurexin, stargazin, and LRRTM2 (Liu et al., 2013; Chamma et al., 2016a, b). Small tags that are detected by nanobodies directly, without the use of biotin, have also been developed recently (for example Virant et al., 2018).

### Visualizing Synapse Volume and Activity

Most labels described above allow to specifically reveal distinct organelles or proteins. To visualize the overall synapse morphology and volume, probes with lower specificity can be used. Calcium imaging is a classical approach, since it does not specifically label any of the synaptic components, but allows visualizing both the synaptic volume and synaptic activity. Synaptic physiology relies heavily on the calcium concentration: in the presynapse it triggers synaptic vesicle exocytosis, while in the post-synapse it regulates synaptic plasticity. Calcium imaging allows monitoring intracellular changes in the calcium concentration by the use of calcium indicators – molecules whose fluorescence changes upon binding to calcium (reviewed in Grienberger and Konnerth, 2012). Calcium indicators can be a useful tool to visualize synapse volume since due to not being specific to any organelles, they fill the whole synapse, effectively illuminating the total synaptic volume. Many calcium indicators with different modes of action exist and can be divided into two groups: genetically encoded and synthetic indicators. The first indicator to be used was the bioluminescent protein aequorin, which emits blue light upon binding to calcium without the need of excitation by light (Shimomura et al., 1962). Multiple different genetically encoded calcium sensors exist (Lin and Schnitzer, 2016). Some, such as Yellow Cameleon, rely on Förster resonance energy transfer (FRET) to function. Yellow Cameleon is a chimeric protein consisting of a calcium-bindng protein calmodulin, a calmodulin-binding peptide and to two fluorescent proteins: ECFP and Venus. Upon binding to calcium, calmodulin undergoes conformational changes that bring ECFP and Venus close enough to enable FRET, effectively shifting emitted light from blue to green (Nagai et al., 2004). The second group of genetically encoded calcium indicators includes proteins with a single fluorophore. One example is GCaMP family of proteins. These proteins consist of EGFP flanked on different sides by calmodulin and a calmodulin-binding peptide. Conformational changes in presence of calcium lead to an increase of the fluorescence intensity (Nakai et al., 2001). Newer genetically encoded calcium sensors have been developed to provide wider color selection and smaller size. Most genetically encoded calcium dyes emit green light, but a few variants with other wavelengths also exist, such as blue B-GECO (Zhao et al., 2011), red R-CaMP2 (Inoue et al., 2015), R-GECO (Zhao et al., 2011), jRCaMP1 and jRGECO1 (Dana et al., 2016), and near-infrared NIR-GECO1 (Qian et al., 2019) and GAF-CaMP2 (Subach et al., 2019). NTnC is a recently developed small and bright calcium indicator that combines the sensing part of FRET sensors with reporting domain of single fluorophore sensors (Barykina et al., 2016). Genetically encoded calcium sensors can be used for calcium imaging in cultured cells following transfection, or in transgenic animals where their expression can be specifically targeted to neural cells and can be maintained over long time periods. However, creating and maintaining such transgenic lines can be time-consuming and expensive. Synthetic indicators are small chemicals that consist of a chelating site which is binding calcium ions, and fluorescent chromophore part which emits light. Examples of such indicators include Quin, Fura, Oregon Green and Fluo calcium dyes. Upon binding to calcium these indicators display changes in fluorescence intensity and/or shift in peak excitation or emission wavelength (Paredes et al., 2008). Ratiometric indicators such as Fura-2 show a change in the excitation wavelength upon calcium binding and allow quantitatively measurements of intracellular calcium concentration that are not affected by the probe concentration. Synthetic calcium dyes are available in a large variety of spectral characteristics and different affinities to calcium (Takahashi et al., 1999), do not require transfection to be delivered in the cells and thus allow for faster experimental procedures, however, are usually expelled from the cells during long experiments and hence are difficult to use for long-term imaging, as well as cannot be targeted to a specific cell type (Paredes et al., 2008). An important point of consideration when using both genetically encoded and synthetic calcium dyes is their possible cytotoxicity. Due to their binding to calcium, calcium sensors act as calcium buffers, effectively decreasing the free calcium concentration in the cells, which can lead to significant changes in the cellular physiology, especially in the context of synaptic activity where calcium plays crucial role (McMahon and Jackson, 2018).

Apart from calcium imaging, multiple approaches were developed to visualize synaptic activity (Lin and Schnitzer, 2016; Deo and Lavis, 2018). These include voltage, neurotransmitter and vesicle fusion sensors. To monitor changes in the membrane potential, both small organic molecules such as cyanine dyes (Miller, 2016) or VoltageFluor (Woodford et al., 2015), and genetically encoded sensors (for example based on a voltage-sensitive phosphatase, St-Pierre et al., 2015) can be used. Molecules that display an increase in the fluorescence intensity upon binding to a neurotransmitter (e.g., ExoSensor, Klockow et al., 2013) or genetically encoded sensors containing a neurotransmitter-binding domains of natural proteins (e.g., FLIPE, Okumoto et al., 2005) are employed to directly detect neurotransmitters. Alternatively, synaptic vesicle exocytosis can be visualized as a measure of synaptic activity. This is achieved through usage of FM dyes or of fluorescent neurotransmitters. The latter mimic natural neurotransmitters, are loaded into the synaptic vesicles, and are released during synaptic activity. By following the fluorescence of these false neurotransmitters one can visualize synaptic vesicle release, just as for the FM dyes (Gubernator et al., 2009).

The most commonly used tools for measuring synaptic release are currently pH-sensitive variants of GFP (pHluorins). Synapto-pHluorin is a pHluorin sensor based on synaptic vesicle protein VAMP2, and is ∼10-fold more fluorescent at neutral pH than in the acidic environment of the synaptic vesicles. Synapto-pHluorin localizes to the synaptic vesicles and emits light only after the vesicle is exocytosed, when the fluorescent protein is exposed to the neutral pH of the extracellular medium (Sankaranarayanan et al., 2000; Figure 1B).

### Visualizing the Synaptic Cytoskeleton: Affinity Tools

**FIGURE 5:**
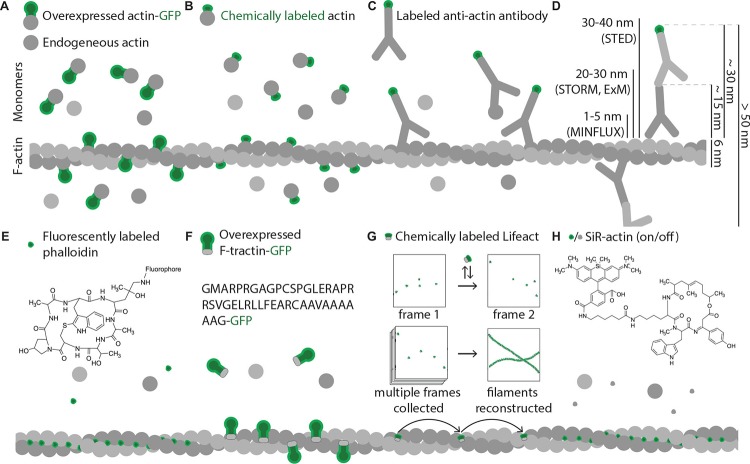
Commonly used actin probes. Green shapes represent fluorescent moieties; all molecules shown in different shades of gray are non-fluorescent and hence are invisible under fluorescent microscope. Size differences of the shapes approximately represent size differences of the molecules. **(A)** Ectopically expressed actin-GFP partially incorporates into actin filaments but also contributes to background fluorescence from monomeric actin-GFP molecules and increases the concentration of the monomers. **(B)** Due to small size of dyes, chemically labeled actin has higher polymerization ability, however, still displays significant background fluorescence from monomers and also increases the concentration of actin monomers. **(C)** Density of antibody labeling depends on epitope accessibility and is significantly restricted by large size of antibody molecules, which also introduces large linkage errors; background fluorescence observed from antibodies bound to actin monomers in solution. **(D)** Comparison of fluorophore displacement from targeted epitope caused by common immunostaining procedures with resolution abilities of modern super resolution methods. When a combination of primary and secondary antibodies is used to image an actin filament, fluorophores of antibodies that recognize neighboring actin subunits might be located more than 50 nm apart (10 times larger distance then a thickness of an actin filament). Modern microscopy techniques can resolve objects that are as close as few nanometers apart, so usage of such large probes leads to significant loss of advantages super-resolution methods can offer. **(E)** Phalloidin is a small chemical that binds exclusively F-actin with high specificity and affinity, shows high density of labeling and low background signal. **(F)** Genetically encoded actin binders (F-tractin illustrated as an example) fused to GFP bind F-actin *in vivo*. Unbound molecules contribute to background fluorescence, but the concentration of actin monomers is not changed. **(G)** Low affinity of Lifeact binding to F-actin can be used for certain types of super resolution microscopy. Here Lifeact coupled to a dye acts as an exchangeable probe. Multiple frames are collected with Lifeact molecules having different locations in different frames. Post-imaging processing allows reconstructing F-actin architecture from all individual Lifeact locations. **(H)** SiR-actin is cell membrane-permeable, specifically labels F-actin and additionally has low fluorescence when not bound to F-actin (off state) but cannot be fixed by aldehydes.

Widely used for other cellular components, GFP fusions and antibody stainings have been less effective for visualization of cytoskeletal filaments. The common major problem is that both approaches result in labeling of both monomers and cytoskeletal filaments (Figures 5A,C), decreasing the apparent signal to noise ratio. This problem is relatively easily solved for antibody stainings in fixed samples by detergent treatments that remove most soluble proteins, but is prominent when overexpression of monomers fused to fluorescent proteins is used, since chimeric proteins are less likely to get incorporated in the filaments (Westphal et al., 1997). As a result, significant fraction of the fluorescence comes from the free monomers, while cytoskeletal filaments are only partially labeled as they mainly consist of native proteins that are not labeled by the fluorescent protein (Figure 5A). To increase polymerization ability and decrease effects of bulky GFP, the monomers can be coupled to small chemical fluorophores instead of overexpression as GFP fusion (Kellogg et al., 1988). This approach requires technically challenging microinjections to be performed to deliver labeled monomers into the cells and does not result in high density of labeling since fluorophore-coupled monomers just like the ones labeled with fluorescent proteins are less likely to polymerize than native endogenous proteins (Figure 5B; Kovar et al., 2006). In addition, both approaches change the native concentration of actin monomers in the cell, while most physiological processes requiring actin polymerization still rely on buffering by endogenous unmodified actin. Multiple effects of GFP fusions on actin dynamics have been reported (Aizawa et al., 1997; Feng et al., 2005; Deibler et al., 2011), rendering live-cell measurements of actin dynamics based on labeled actin monomers to some extent unrepresentative of the physiological situation. Nonetheless, it remained to be one of the most popular approaches to visualize actin in live cells for years since it’s easy to perform, and many insights in cytoskeleton dynamics were obtained by utilizing fusions with fluorescent proteins (Doyle and Botstein, 1996; Westphal et al., 1997; Lorenz et al., 2004; Dovas et al., 2009, 2011; Flynn et al., 2009; Oser et al., 2009; Francis et al., 2011; Koskinen and Hotulainen, 2014; Lei et al., 2017).

To make GFP-based labeling more specific to cytoskeletal structures, filament-binding proteins can be targeted instead of cytoskeletal proteins themselves. This is a common approach to label F-actin and GFP-fusions of actin-binding domains are often used to reveal actin cytoskeleton. Examples of such domains will be discussed in detail later. Similarly, to track microtubule dynamics, it is common to label plus-end-tracking proteins with GFP instead of tubulin itself (Stepanova et al., 2003; Yau et al., 2016). Also, targeting with antibodies native proteins that bind cytoskeletal elements, but do not form filaments, can be useful for the visualization of some features of cytoskeleton. Thus, anti-spectrin antibodies are commonly used for visualization of the neural membrane-associated periodic skeleton (Xu et al., 2013; D’Este et al., 2015, 2016; He et al., 2016; Vassilopoulos et al., 2019), and expression of spectrin-GFP allowed to visualize this structure in live cells (Zhong et al., 2014).

While immunostained microtubules can be imaged well with diffraction limited imaging techniques, many observations of microtubules with super resolution microscopy revealed that antibodies are not able to provide sufficiently high quality of staining. The density of labeling achieved by antibodies is low enough for modern imaging techniques to resolve single fluorophores along the microtubules. This results in a single microtubule appearing as a row of separate aligned objects instead of one continuous filament or creates an impression of microtubules being interrupted (Bossi et al., 2008; Huang et al., 2008; Heilemann et al., 2009; Wildanger et al., 2009a; Gould et al., 2011). The same is also observed in the case of neurofilaments (Hell, 2007; Wildanger et al., 2009b) which are in some cases cannot even be seen as rows of aligned objects but rather a set of randomly placed dots, making it impossible to make any conclusions about neurofilament cytoskeleton architecture (Donnert et al., 2006; Rankin et al., 2008). Even conventional imaging can show that most neurofilament antibodies do not provide a high quality of staining. In contrast, many anti-tubulin antibodies are known to have high affinity and specificity for tubulin. Their low labeling density can be attributed to the large size of the antibodies, which does not allow them to bind many of the epitopes, due to steric hindrance.

Another crucial drawback of the antibodies caused by their large size is the fact that they introduce a substantial linkage error what makes them unsuitable for super-resolution studies of such fine structures as cytoskeletal filaments. Actin filaments, for example, have a diameter of ∼5 nm, while conventional antibodies have a size of ∼15 nm, placing the fluorophore at a several-fold larger than the thickness of the filament itself distance from the filament. With recent super resolution microscopy methods offering nanometer resolution (Balzarotti et al., 2017), the usage of such large labels might lead to erroneous conclusions about protein locations and the shapes and sizes of the structures under investigation (Figure 5D). For example, based on electron microscopy observations, microtubules are known to have a diameter of 25 nm (Theg, 1964). After a conventional immunostaining procedure, the perceived thickness of the microtubules is increased approximately 2.5-fold due to the size of primary and secondary antibodies now decorating the microtubules. While this increase is not noticeable in diffraction limited microscopy since the resulting thickness is still less than 200 nm, it can be observed using super resolution technics and can be substantially reduced if smaller labels are used (Ries et al., 2012; Pleiner et al., 2018). Also, co-localization of proteins can be underestimated at high resolutions (Xu et al., 2016), an effect that would be increased when additional linkage error is introduced by antibodies, as has been shown for SNAP25 and syntaxin 1 clusters (Maidorn et al., 2019). Additionally, antibodies cannot be used for live imaging of cytoskeleton unless delivered through microinjections.

As an alternative to classical antibodies, derivatives of single chain camelid antibodies – nanobodies – can be used. Their considerably smaller size (<5 nm) makes nanobodies a better probe to be used with super resolution microscopy as they allow to overcome many of the problems discussed above. Since nanobodies consist of only one protein chain, they can also be fused to fluorescent proteins and expressed in cells, allowing live cell imaging. A commercially available Actin-Chromobody^®^ (Chromotek, Germany) have been used to track actin dynamics in plants, zebrafish, and nuclei of mammalian cells (Rocchetti et al., 2014; Panza et al., 2015; Plessner et al., 2015) as well as for super resolution live imaging of actin in neurons (Wegner et al., 2017) and for correlative light electron microscopy, where chromobodies were labeled with anti-GFP and gold-conjugated secondary antibodies (Abdellatif et al., 2019). A stable cell line expressing Actin-Chromobody has been generated, allowing tracking actin dynamics without the need of transfections (Keller et al., 2019). While ectopic expression of fluorescent protein leads to relatively high background coming from the unbound molecules, Actin-Chromobodies offer an advantage over direct actin-GFP fusion since they do not increase the total amount of actin molecules in the cell and report localization and dynamics of endogenous actin. Nevertheless, some studies report that at high expression levels Actin-Chromobodies can affect actin morphology in neurons (Wegner et al., 2017), presumably due to the chromobodies modifying either the dynamics of monomeric actin, by increasing the mass and size of the molecule, or the polymerization process, through steric hindrance. Synthetic anti-actin nanobodies have been also developed for use in staining of fixed cells (Moutel et al., 2016), however, their performance in super resolution imaging was not tested, and they are only compatible with methanol fixation – a fixation method that is usually avoided when actin cytoskeleton is targeted, and often destroys epitopes for classical antibodies, making co-immunostaining with other proteins challenging.

Anti-tubulin nanobodies have also been developed, allowing to visualize some structures that cannot be resolved using conventional antibodies at all. The spacing between microtubules in densely packed bundles, which are found in axons, is ∼20–70 nm (Chen et al., 1992). When microtubules are labeled with antibodies each having size of 15 nm, signals from fluorescent labels on antibodies merge together. This does not allow resolving individual microtubules in such bundles despite high resolution power of modern microscopes – a problem that has been solved with application of anti-tubulin nanobodies (Mikhaylova et al., 2015). Chemically labeled anti-tubulin nanobodies allow nanometer resolution in fixed cells (Mikhaylova et al., 2015; Fabricius et al., 2018), but have not been tested in live cells as fusions to fluorescent proteins so far.

As an alternative to nanobodies, affimers, which are similar in size, can be also used. Affirmers binding both actin and tubulin were recently developed, allowing visualization of these cytoskeletal elements in fixed cells and *in vivo* (Tiede et al., 2017; Lopata et al., 2018).

### Visualizing the Synaptic Actin Cytoskeleton: Actin- and Tubulin- Specific Toxins and Other Small Labels

While immunostainings and GFP fusions remain to be the main approaches to visualize some of the cytoskeletal components such as neurofilaments and septin filaments, multiple small probes were developed to overcome above-described difficulties associated with direct actin coupling to fluorophore or immunostainings to make both live and super resolution imaging of actin cytoskeleton possible.

A classical tool for actin labeling is phalloidin – a toxin from *Amanita phalloides* that binds F-actin and prevents actin depolymerization (Wulf et al., 1979). Phalloidin is a small cyclopeptide with a size of ∼6 Å, it has high affinity and specificity to actin filaments, shows no binding to actin monomers, and provides high labeling density and signal to noise ratio in fluorescence microscopy (Figure 5E). It has been used for actin visualization for decades and majority of information on actin distribution in neurons was obtained from phalloidin stainings (Drenckhahn et al., 1984; Bernstein and Bamburg, 1992; Morales et al., 2000; Shupliakov et al., 2002; Bleckert et al., 2012; Blunk et al., 2014). It is a great choice for super resolution light microscopy when labeled with suitable fluorophore as been shown by multiple groups in recent years that used it in STED (Mace and Orange, 2012; D’Este et al., 2015; Neupane et al., 2015; Bär et al., 2016; Sidenstein et al., 2016), single molecule localization microscopy including STORM and dSTORM (van den Dries et al., 2013; Xu et al., 2013; Ganguly et al., 2015; Leyton-Puig et al., 2016; Hauser et al., 2018; Pan et al., 2018), and structured illumination microscopy (Guizetti et al., 2011; Zobel and Bogdan, 2013). It cannot, however, be used for live cell experiments as it does not readily penetrate the plasma membrane, is toxic if delivered intracellularly, and alters actin polymerization even at small concentrations, making studies of actin dynamics impossible (Wehland et al., 1977; Coluccio and Tilney, 1984; Cooper, 1987; Visegrády et al., 2004).

Toxin-based labels are also used for microtubules visualization. Paclitaxel (also known as taxol), a drug that induces tubulin assembly (Caplow et al., 1994), is one such example. It can be used as a constantly fluorescent derivative (Abal et al., 2001; Lillo et al., 2002; Barasoain et al., 2010) or as a modified reagent that only attains fluorescence inside the cell – known as Tubulin Tracker^TM^ (Thermo Fisher Scientific), available with green and deep-red fluorophores (Grau et al., 2013; Zarrouk et al., 2015; Gao et al., 2018; Wang et al., 2018). These derivatives are membrane-permeable and can label microtubules in live cells when simply added to the cellular medium, but cannot be used in fixed samples or for long-term imaging as they are not retained well inside the cells. A similar taxol-based probe ViaFluor (Biotum) allows imaging for up to 72 h, and is available with SIM and STED-compatible fluorescent label.

Newer actin labels make live imaging possible precluding mentioned above problems associated with use of direct actin labeling. One common approach is usage of genetically encoded actin labels based on actin-binding proteins. These labels typically consist of an actin-binding domain of a naturally occurring protein fused to a fluorescent protein and can be expressed in the cell allowing tracking of actin filaments in live. GFP-labeled actin-binding domains have a number of advantages over direct actin-GFP fusions or GFP-nanobodies: they predominantly bind to actin filaments and not actin monomers, allowing to visualize the filaments with less background; do not impair actin polymerization as toxins targeting actin do, better preserving native cytoskeleton architecture and allowing to study its dynamics; and do not change the total concentration of actin monomers in the cell, what could otherwise affect cellular physiology through sequestering of actin-binding proteins, initiation of polymerization or other mechanisms (Figure 5F). Three such labels became popular in recent years: UtrCH, F-tractin, and Lifeact (Schell et al., 2001; Burkel et al., 2007; Riedl et al., 2008). UtrCH, a label consisting of the first 261 amino acids of human actin-binding protein Utrophin and a fluorescent protein, does not stabilize F-actin *in vitro* (Burkel et al., 2007) and have been used for live imaging of actin in neurons (Ganguly et al., 2015; Balasanyan et al., 2017). F-tractin, a 43 amino acids long fragment of rat inositol 1,4,5-trisphosphate 3-kinase A (Johnson and Schell, 2009), has also been used for live imaging in neurons (Johnson and Schell, 2009; Merriam et al., 2013; Winans et al., 2016) as well as other cell types (Yi et al., 2012).

Lifeact, derived from yeast F-actin-binding protein Abp140, is the most commonly used genetically encoded actin label. Unlike other actin-binding domains, Lifeact does not have homologs in higher eukaryotes, and is also the smallest of the available genetically encoded labels, consisting of only 17 amino acids (Riedl et al., 2008), which contributed to the growing popularity of this label. Lifeact has been extensively used for live imaging in various cell types including neurons (Li et al., 2008; Vidali et al., 2009; Jang et al., 2010; Römer et al., 2010; Deinhardt et al., 2011; Dupin et al., 2011; Huang et al., 2012; Kerr and Blanpied, 2012; Taylor et al., 2012; Merriam et al., 2013; Torregrosa-Hetland et al., 2013; Kronlage et al., 2015; Lu et al., 2015), as well as for super resolution, including live-PALM (Fuchs et al., 2010; Izeddin et al., 2011), live-RESOLFT and STED of living brain slices (Urban et al., 2011; Testa et al., 2012), and structured illumination microscopy (Rego et al., 2012; Li et al., 2015). Transgenic mice expressing Lifeact fused to mRFPruby2 or EGFP were also generated, allowing live studies of actin dynamics without the need of transfections (Riedl et al., 2010). Lifeact can be used for super-resolution microscopy in both live and fixed cells. For live imaging it is usually fused to a far-red fluorescent protein (e.g., mNeptune2) and expressed in neurons for subsequent visualization with live super resolution techniques (Urban et al., 2011; Testa et al., 2012; Wegner et al., 2017). To achieve even higher resolution, instead of ectopic expression with a fluorescent protein, purified Lifeact can be labeled with a chemical dye and used for staining of fixed and permeabilized cells. Since Lifeact has low affinity to F-actin (Riedl et al., 2008), its transient association with actin filaments can be visualized with single molecule localization techniques based on probe exchange such as IRIS (Kiuchi et al., 2015; Figure 5G). Low affinity of purified Lifeact binding to F-actin makes it unsuitable for usage in stainings of fixed cells in combination with many other super resolution techniques, since most of them do not rely on probe exchange, but require strong binding to the structure of interest.

Although the small labels discussed here are not expected to have such drastic effects on F-actin morphology and dynamics as actin overexpression or phalloidin, their potential influence on cytoskeletal dynamics must be considered, as growing evidence suggests that they do affect some of the aspects of actin physiology. UtrCH, for example, has been shown to perturb actin assembly dynamics *in vitro* (Belin et al., 2013), increase dendritic branching in cultured neurons (Patel et al., 2017), cause cortical actin breakdown and female sterility during *Drosophila* oogenesis (Spracklen et al., 2014), and stabilize vesicle-actin network in oocytes if expressed at high levels (Holubcová et al., 2013). Its short variant (Utr230) can induce the formation of various actin aggregates in both cell nuclei and cytoplasm (Du et al., 2015). The latter work has reported that Lifeact can also induce actin polymerization, albeit this effect was restricted to cell nuclei and resulted only in filamentous arrangements (Du et al., 2015). Other studies showed that Lifeact has concentration-depended effects on actin nucleation, elongation and cofilin-induced severing, as well as on the length of neurites, dendritic spine morphology and overall morphology of mesenchymal stem cells (Courtemanche et al., 2016; Patel et al., 2017; Wegner et al., 2017; Flores et al., 2019). These effects differ depending on position and identity of the fused fluorescent reporter, the promoter used, and the resulting protein abundance (Courtemanche et al., 2016; Patel et al., 2017; Flores et al., 2019). F-tractin has been reported to induce formation of long filopodia and to perturb the overall morphology of *Xenopus* XTC cells (Belin et al., 2014) and cause abnormal spine elongation (Johnson and Schell, 2009), but did not alter actin dynamics during *Drosophila* oogenesis (Spracklen et al., 2014). Both Lifeact-mEGFP and F-tractin-EGFP, expressed under control of the CMV promoter, have only minor effects on neuronal morphology in primary hippocampal neurons (Patel et al., 2017). Transgenic mice expressing Lifeact were viable and had a normal phenotype, with the primary neurons derived from these mice also demonstrating normal development and morphology (Riedl et al., 2010).

To avoid problems caused by fluorescent protein fusions and protein overexpression, membrane-permeable cytoskeleton labels can be used, such as SiR-actin and SiR-tubulin. SiR-actin is one of the newest probes developed that can be used for live imaging of actin without the need of transfection. It is a silicon-rhodamine based derivative of an actin filament-stabilizing toxin jasplakinolide. It has minimal cytotoxicity, permeates the plasma membrane, and shows an ∼100-fold increase in fluorescence intensity upon binding to F-actin (Figure 5H; Lukinavičius et al., 2014). SiR-actin has been used in a number of studies focused on super resolution imaging of actin cytoskeleton in neurons (D’Este et al., 2015, 2016; Gokhin et al., 2015; Qu et al., 2016; Hoyle et al., 2017) and appears to be the easiest tool to label actin so far: its usage does not require transfection, cell membrane permeabilization or other manipulations to deliver the probe in the cell. A conjugate of silicon-rhodamine and microtubule-stabilizing drug docetaxel, named SiR-tubulin (Lukinavičius et al., 2014), can be used for visualization of microtubules (Robison et al., 2016; Dmitrieff et al., 2017; Magliocca et al., 2017; Larsson et al., 2018; Paknikar et al., 2019). Other similar fluorogenic probes based on STED-compatible dyes (such as 510R, 580CP, GeR) and tubulin-binding drugs cabazitaxel and larotaxel have been also developed recently (Lukinavičius et al., 2018). Fluorogenic character of these labels allows using them without any washing steps, and their spectral characteristics and high photostability make them suitable for super resolution imaging such as STED. While originally described to have low cytotoxicity, SiR-actin and SiR-tubulin are derivatives of F-actin- and microtubule-stabilizing compounds, hence their potential effects on cytoskeleton dynamics and morphology should be thoroughly investigated before they can be considered safe to use for studying actin and tubulin dynamics *in vivo*. Additionally, since SiR-actin and SiR-tubulin have no primary amines it their structures, it is not possible to fix them with commonly used aldehyde-based fixatives, what makes their use in co-immunostainings with other proteins problematic.

In addition to all the individual disadvantages of described probes, they are usually not able to stain all cytoskeletal structures. For example the actin “gold standard” phalloidin as well as Lifeact are not able to bind actin polymers decorated with actin-binding protein cofilin such as for example stress-induced fibers (McGough et al., 1997; Munsie et al., 2009; Sanders et al., 2013). Both Lifeact and actin-GFP label actin cytoskeleton in lamellipodia, but not in filopodia or lamella (Belin et al., 2014). Interestingly, in mesenchyme cells Lifeact only labels proximal regions of the cytoplasmic protrusions, but not the distal tips (Sanders et al., 2013). UtrCH, on the contrary, binds to filaments in lamella and much less in lamellipodia (Belin et al., 2014). While UtrCH is excluded from Arp2/3-induced structures, GFP-tagged actin is often excluded from formin-generated filaments (Chen et al., 2012; Belin et al., 2014). Utr230, a short variant of UtrCH, predominantely binds to the most stable actin structures such as stress-induced fibers and cortical networks, and also stains structures that cannot be visualized with phalloidin such as Golgi-associated filaments (Belin et al., 2014). Anti-actin affimers demonstrate differences in their affinity to stable or dynamic actin filaments (Lopata et al., 2018). Importantly, fluorescent reporters and their positions can also affect the structures that probes can bind (Lemieux et al., 2014), Thus, mEGFP-Lifeact visualized lamellipodia well, while TagRFP-Lifeact is excluded from the same structures (DesMarais et al., 2019). Even small dyes attached to the classical phalloidin can change the quality of staining. For example, staining with Phalloidin-Alexa Fluor^®^ 488 results in more detailed labeling than with Phalloidin-iFluor^TM^ 405, Phalloidin – Alexa Fluor^®^ 488, Phalloidin – Alexa Fluor^®^ 555 or Phalloidin – Alexa Fluor^®^ 647 (DesMarais et al., 2019). Similarly, different clones of commonly used antibodies recognize different populations of actin cytoskeleton (DesMarais et al., 2019). Taken together, this illustrates a strong influence of multiple factors on labels ability to recognize cytoskeletal structures. Consequently, differences in the preferences of the labels for actin structures decorated with certain actin binding proteins should be considered in relation to actin-binding proteins distribution. For example, it is known that in dendritic spines the actin cytoskeleton forms a stable core in the center of the spine, and a more dynamic shell at the periphery. The dynamics of the latter shell is maintained by the actin-depolymerizing factor cofilin, while actin branching Abp2/3 complex is localized closer to the stable core (Rácz and Weinberg, 2013). This differential distribution of actin binding proteins would result in significantly different staining patterns produced by UtrCH and Lifeact, which are excluded from Arp2/3- or cofilin-bound structures, respectively. It is highly likely that less commonly used actin probes, such as F-tractin and SiR-actin, also reveal only a subpopulation of the actin cytoskeleton, and therefore the choice of a label would often depend on specific actin cytoskeleton components one wants to investigate.

### Problems and Solutions in Visualizing the Cytoskeleton in Fixed Cells

Apart from a choice of label, another crucial issue in cytoskeleton visualization is preservation of its structure in fixed cells. While thick stress fibers are preserved well by most fixatives, many fine components of actin cytoskeleton are sensitive to physical and chemical perturbations and are damaged, destroyed or not completely preserved by commonly used fixation procedures. Paraformaldehyde has been shown not to be able to preserve thin actin bundles and structures (Whelan and Bell, 2015; Bachmann et al., 2016) and 0.5–3% glutaraldehyde is commonly used as fixative to preserve actin cytoskeleton, as it provides more effective cross-linking. Nonetheless, even after fixation with glutaraldehyde, actin cytoskeleton can still be severely damaged by following procedures routinely used for visualization of other structures such as osmium tetroxide staining for transmission electron microscopy (Maupin-Szamier and Pollard, 1978), highlighting the need for careful choice of treatment procedures when imaging actin cytoskeleton. To stabilize actin cytoskeleton, specific buffers containing MgCl_2_and EGTA are used during fixation (Small, 1988). Additionally, most cytoskeleton fixation procedures involve extraction of all other cellular components before strong fixation of the filaments. This is usually done by adding relatively high amounts on detergents and leads to loss of all cellular membranes and most soluble proteins (Rinnerthaler et al., 1988; Xu et al., 2013). This results in better actin staining and reduced background signal, however, makes in very difficult to image actin cytoskeleton at the same time with other proteins as (a) most proteins are washed away after the extraction and (b) many conventional antibody epitopes get destroyed by glutaraldehyde fixation significantly reducing the effectiveness of immunostainings. Paraformaldehyde fixation in actin-stabilizing buffer with no extraction can be used when actin co-staining with other proteins is required. This results in less effective preservation of actin ultrastructure, but might still be sufficient to visualize certain structures (Xu et al., 2013). In addition to actin-stabilizing buffers, paraformaldehyde fixation can be further improved if performed at 37°C, illustrating that temperature can also affect the quality of fixation (Leyton-Puig et al., 2016; Pereira et al., 2019). In general, the choice of fixation procedure, just like the choice of a label to use, still largely depends on structures one aims to image and other specific requirements of the experiments and no universal method have been developed yet (Richter et al., 2017).

### Combining Genetic Encoding With Chemical Labeling: Enzymatic Tagging and Click Chemistry

As described above, usage of both genetically encoded labels and chemically labeled probes have some disadvantages. The most prominent ones are low fluorescence intensity of fluorescent proteins and often high background/non-specific binding of affine probes. To solve these problems, techniques combining genetic encoding and chemical labeling have been developed. These techniques usually involve genetic manipulation of the protein of interest, resulting in attachment of a tag sequence to it. This tag is then specifically recognized and covalently bound to chemical fluorophores of choice. This results in highly specific labeling of only proteins containing the tag with highly fluorescent chemical fluorophores. Two examples where such an approach is used are SNAP and Halo tags. The SNAP tag is a 182 amino acids long polypeptide that can be fused to a protein of interest, generating a chimeric protein that is not fluorescent. The SNAP tag is derived from O^6^-alkylguanine-DNA alkyltransferase, whose natural function is to transfer the alkyl group of O^6^-alkylated guanine in DNA to a cysteine residue in the alkyltransferase active center after which the alkyltransferase is permanently inactivated. Mutations introduced to generate SNAP tag changed its specificity to benzylguanine derivatives of type 1 (Keppler et al., 2003), which can be generated from many common fluorophores. When such fluorescent derivatives are added to cells expressing SNAP tag, the latter catalyzes self-labeling with the fluorophore by covalently attaching the fluorophore with the benzyl group to a cysteine residue in SNAP tag sequence. The reaction is highly specific and can be highly effective, resulting in nearly all present SNAP tags labeling, but recent studies report much lower efficiency (Thevathasan et al., 2019). The labeling reaction can be triggered in live cells when membrane permeable dyes such as tetramethyl-rhodamine-Star or 647-SiR are used. The HaloTag is a similar self-labeling polypeptide generated from bacterial haloalkane dehalogenase (Los et al., 2008). Similar to SNAP tag, HaloTag it is a non-fluorescent tag that catalyzes transfer of reactive compounds (species modified to be recognizable by the HaloTag – HaloTag ligands) to itself (Los et al., 2008). Available HaloTag ligands include cell-permeable dyes tetramethylrhodamine, diacetyl derivative of fluorescein, rhodamine 110 and Oregon Green, as well as cell-impermeable ones Alexa Fluor^®^ 488 and Alexa Fluor^®^ 660.

While these tags provide fast, specific and efficient protein labeling in cells, an obvious disadvantage is rather large size of the tags (19.4 and 33 kDa for SNAP and Halo tags, respectively). Similar to labeling with fluorescent proteins, in some cases attachment of such a tag might impede the natural targeting and trafficking of the protein. This problem can be solved by substituting a large polypeptide tag with a small moiety, which can interact quickly and specifically with another small compound, effectively conjugating them. Such process is referred to as click chemistry and in general is represented by multiple reactions with different mechanisms. One example of such a reaction is copper-mediated azide–alkyne cycloaddition. It can be used to specifically label proteins with chemical dyes without the need to introduce a large tag. Instead, a single amino acid containing an alkyne group has to be incorporated in the protein of interest. A dye with an azide moiety can be added to the specimen and bound covalently to the alkyne, labeling the protein with the fluorophore (Milles et al., 2012). While offering an advantage of a small tag that should not interfere with protein’s localization and functioning, this is a rather challenging and labor-demanding approach. Since it requires the presence of unnatural amino acids in the protein of interest, a relatively complicated genetic setup has to be used to provide machinery for inclusion of the unnatural amino acid in the normal protein translation.

## Conclusion

Over the years multiple methods and approaches were developed to label synaptic organelles and structures (Table 1). Many of these rely on naturally evolved compounds such as intrinsically fluorescent proteins or natural toxins, while others employ rational design and chemical synthesis or modifications. Respectively, they all have their own advantages and preferred uses, and none of the available labels suits every experiment. The first point of consideration should always be whether selected label can introduce biological artifacts that would lead to erroneous conclusions. For example, while the use of GFP-actin chimeras might not result in high signal to noise ratio when imaging actin dynamics, it would still be a preferred method compared to use of toxins altering actin dynamics, such as phalloidin. This would not be a problem when fixed cells are imaged. At the same time, the optical characteristics of GFP might not be suitable for some super resolution techniques, and chemically labeled phalloidin would be preferred there. Similarly, while quantum dot-conjugated antibodies provide high specificity and photostability, the size of the quantum dots might limit the structures that can be effectively visualized. Although specificity of labeling is the main concern when imaging synaptic organelles, the compatibility of the labels with specimen preparation should be also carefully considered. This is an especially important point when imaging cytoskeletal elements, as those are not preserved by many commonly used procedures. With the wide selection of different labels for synaptic organelles, the perfect use still depends on specific experimental requirements and novel imaging techniques often require novel probes to be developed.

**TABLE 1:** Probes to specifically label synaptic structures and their potential uses.

							**Compatible with**	**Membrane-**
**Label**	**Specificity**	**Chemical nature**	**MW/size**	**Live imaging**	**Super resolution**	**EM**	**aldehyde fixation**	**permeable**
Antibodies	Almost any protein	Multi-chain proteins	∼150 kDa	Only for proteins exposed on the PM surface	Yes, but reduce performance of techniques capable of resolution <40 nm	Yes	Yes	No
FM1–43	PM, recycling membranes	Styryl dye	0.61 kDa	Yes	No	Yes	No	No
FM1-43FX	PM, recycling membranes	Styryl dye	0.56 kDa	Yes	No	Yes	Yes	No
mCLING	PM, recycling membranes	Palmitoylated octapeptide	1.2 kDa	Yes	Yes	No	Yes	No
DMPE-cypHer5E	Membranes of acidic organelles	Phospholipid conjugated to pH-sensitive organic dye	1.4 kDa	Yes	No	No	No	No
Quantum dots	Depends on antibody/streptavidin coating, can be directed to PM proteins or luminal domains of vesicular proteins	Inorganic semiconductor nanocrystals, have to be covered with layers of organic molecules	10–40 nm	Yes	Yes	Yes	Yes	No
Acridine orange	Lysosomes	Fluorescent cationic dye	0.3 kDa	Yes	No	No	No	Yes
DAMP	Lysosomes	Non-fluorescent weakly basic amine	0.4 kDa	No	Yes, when labeled by antibodies	No^∗∗^	Yes	Yes
LysoTracker	Lysosomes	Fluorophore linked to a weak base	0.4 kDa	Yes	Difficult	Yes	No	Yes
Rhodamine 123	Mitochondria	Membrane-potential-sensitive organic dye	0.4 kDa	Yes	Difficult	No	No	Yes
TMRM	Mitochondria	Membrane-potential-sensitive dye	0.5 kDa	Yes	Yes	No	No	Yes
TMRE	Mitochondria	Membrane-potential-sensitive dye	0.5 kDa	Yes	Difficult	No	No	Yes
MitoTracker	Mitochondria	Membrane-potential-sensitive dye with a thiol-reactive moiety	0.5 kDa	Yes	Yes (for red-shifted variants)	No	Yes	Yes
ER-Tracker	ER	Small fluorescently labeled organic molecule	∼1 kDa	Yes	Yes (for red-shifted variants)	No	Partially	Yes
ER thermo yellow	ER	Small fluorescently labeled organic molecule	0.6 kDa	Yes	No	No	Yes	Yes
NH_2_-BODIPY	ER	Small fluorescently labeled organic molecule	0.5 kDa	Yes	Yes	No	Yes	Yes
Certain neurotoxins	Various post-synaptic receptors	Peptides and short proteins	1.3–10 kDa	Yes	Yes	No	Yes	No
Actin-Chromobody	Actin	Anti-actin nanobody fused to a fluorescent protein	42 kDa	Yes	Yes	No^∗∗^	Yes	No, but can be expressed in the cells
hs2dAb anti-actin	Actin	Synthetic single domain antibody	14 kDa	No	Yes	No	No	No
Anti-tubulin nanobody	Tubulin	Camelid single domain antibody	∼14 kDa	Potentially yes^∗^	Yes	No	Yes	No
Phalloidin	F-actin	F-actin-stabilizing toxin	0.8 kDa	No	Yes	No^∗∗^	Yes	No
Tubulin Tracker	Microtubules	Fluorescently labeled mictotubule-stabilizing toxin	∼1.3 kDa	Yes	No	No	No	Yes
ViaFluor	Microtubules	Fluorescently labeled mictotubule-stabilizing toxin	∼1.3 kDa	Yes	Yes	No	No	Yes
UtrCH	F-actin	Actin-binding domain of Utrophin	261 aa	Yes	Potentially yes^∗^	No	Yes	No, but can be expressed in the cells
F-tractin	F-actin	Actin-binding domain of inositol 1,4,5-trisphosphate 3-kinase A	43 aa	Yes	Potentially yes^∗^	No	Yes	No, but can be expressed in the cells
Lifeact	F-actin	Actin-binding domain of Abp140	17 aa	Yes	Yes	No	Yes	No, but can be expressed in the cells
SiR-actin	F-actin	Fluorogenic derivative of an actin filament-stabilizing toxin	∼1.3 kDa	Yes	Yes	No	No	Yes
SiR-tubulin	Microtubules	Fluorogenic derivative of a microtubule-stabilizing toxin	∼1.3 kDa	Yes	Yes	No	No	Yes

*^∗^The nature of the label is compatible with the corresponding method, but its performance has not been tested yet; ^∗∗^the label itself is not visible in EM, but can be visualized with gold-coupled antibodies against the fluorophore (either organic dye or fluorescent protein).*

## Author Contributions

Both authors took part in designing and writing the manuscript.

## Conflict of Interest Statement

The authors declare that the research was conducted in the absence of any commercial or financial relationships that could be construed as a potential conflict of interest.
